# Assessing Modeled CO_2_ Retention and Rebreathing of a Facemask Designed for Efficient Delivery of Aerosols to Infants

**DOI:** 10.5402/2012/721295

**Published:** 2012-06-26

**Authors:** Christian Mundt, Alexander Sventitskiy, Jeffrey E. Cehelsky, Andrea B. Patters, Markus Tservistas, Michael C. Hahn, Gerd Juhl, John P. DeVincenzo

**Affiliations:** ^1^Faculty of Aeronautics and Astronautics, Institute for Thermodynamics, University of Bundeswehr Munich, 85579 Neubiberg, Germany; ^2^Alnylam Pharmaceuticals Inc., Cambridge, MA 02142, USA; ^3^LeBonheur Children's Hospital, Memphis, TN 38103, USA; ^4^Department of Pediatrics, University of Tennessee Health Science Center, Memphis, TN 38103, USA; ^5^PARI Pharma GmbH, 82166 Gräfelfing, Germany; ^6^Quintiles Ltd., 80639 Munich, Germany; ^7^Department of Molecular Sciences, University of Tennessee Health Science Center, 50 N. Dunlap, Memphis, TN 38103, USA

## Abstract

*Background*. New aerosol drugs for infants may require more efficient delivery systems, including face masks. Maximizing delivery efficiency requires tight-fitting masks with minimal internal mask volumes, which could cause carbon dioxide (CO_2_) retention. An RNA-interference-based antiviral for treatment of respiratory syncytial virus in populations that may include young children is designed for aerosol administration. CO_2_ accumulation within inhalation face masks has not been evaluated. *Methods*. We simulated airflow and CO_2_ concentrations accumulating over time within a new facemask designed for infants and young children (PARI SMARTMASK^®^ Baby). A one-dimensional model was first examined, followed by 3-dimensional unsteady computational fluid dynamics analyses. Normal infant breathing patterns and respiratory distress were simulated. *Results*. The maximum average modeled CO_2_ concentration within the mask reached steady state (3.2% and 3% for normal and distressed breathing patterns resp.) after approximately the 5th respiratory cycle. After steady state, the mean CO_2_ concentration inspired into the nostril was 2.24% and 2.26% for normal and distressed breathing patterns, respectively. *Conclusion*. The mask is predicted to cause minimal CO_2_ retention and rebreathing. Infants with normal and distressed breathing should tolerate the mask intermittently delivering aerosols over brief time frames.

## 1. Introduction

New aerosol drugs may require more efficient delivery systems, but devices to safely and effectively deliver aerosols to young children have been insufficiently evaluated. Because very young infants are obligate nose breathers, they cannot receive aerosols via a mouthpiece and therefore require a facemask. Both the contour of the mask [1] and the seal between the mask and the patient's face affect aerosol delivery. Leakage between the face and the mask reduces the drug delivery to the patient [2, 3] and increases facial deposition [3]. Thus, a relatively tight seal between the mask and the face is favorable to maintain a high-percent delivered dose. However, a tightly sealed mask increases the dead space, as CO_2_-enriched exhaled breath only partially escapes the mask. Infants have low tidal volumes (~6 mL/kg body weight). Thus, during prolonged dosing of an aerosol, exhaled CO_2_ might accumulate within a tight-fitting mask and form a large component of rebreathed air. Most commercially available inhalation facemasks for infants are intended for metered dose or dry powder inhalers, which require only very short dosing (2-3 breaths). Such short dosing avoids possible CO_2_ accumulation. Other inhalation facemasks for infants are designed for jet nebulizers, which generate the aerosol with a forced column of air or oxygen. This column of air carrying the aerosol flows past the infant's face through a loose-fitting mask, thereby washing the CO_2_-enriched air out of the mask. Thus, with jet nebulization, aerosol delivery efficiency may not be as high as with vibrating membrane nebulizers, but CO_2_ accumulation, although not evaluated, is not thought to be of concern. Unlike jet nebulizers, vibrating membrane nebulizers are not operated with a driving air column. This offers the advantage of higher and more efficient drug delivery to the lung.

Every year in the USA an estimated 2.1 million children less than five years of age receive medical attention because of respiratory syncytial virus (RSV) infection [4]. Most of these infections occur in previously healthy children, and the rates of infection are highest in children less than one year of age [4]. The numbers of outpatient visits (emergency department visits plus pediatric practice visits) for RSV infection in children less than six months of age are estimated at approximately 200/1000 patients [4]. Alnylam Pharmaceuticals (Cambridge, MA, USA) and Cubist Pharmaceuticals (Lexington, MA, USA) are developing an RNA-interference-based antiviral drug (ALN-RSV) for the treatment of RSV in certain populations [5–7]. ALN-RSV is designed to be administered directly into the respiratory tract via an aerosol.

The PARI SMARTMASK^®^ Baby [8] (PARI Pharma GmbH, Starnberg, Germany) has been designed for use with nebulizers based on eFlow^®^ technology, which utilizes a perforated vibrating membrane to generate the aerosol. In order to determine the basic gas flow and CO_2_ distribution inside the PARI SMARTMASK^®^ Baby, the flow inside the mask was analyzed from a fluid dynamic perspective. We focused on the concentration of CO_2_ over time during cycles of modeled infant respiration. The mathematical modeling and resulting CO_2_ concentrations are examined followed by a discussion of the medical relevance to infants. The analysis was conducted in two steps. A preliminary analysis with relatively crude simplifications was undertaken first. Then, the 3-dimensional unsteady computational fluid dynamics (CFDS) simulation was evaluated.

## 2. Methods

### 2.1. Determination of Mask Features

**Figure 1:**
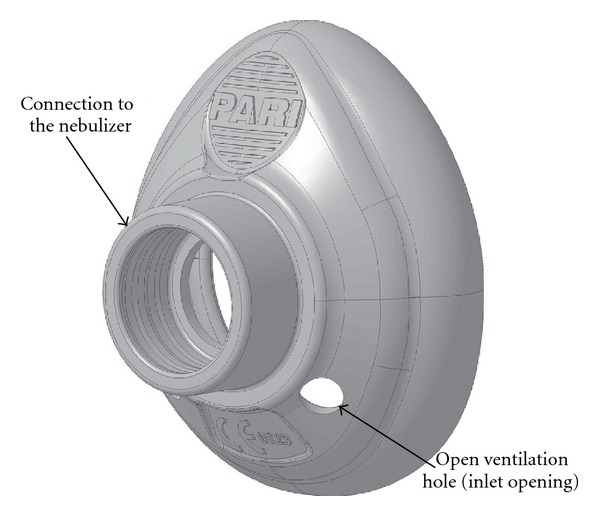
Solid model of the PARI SMARTMASK^®^ Baby.

The PARI SMARTMASK^®^ Baby facemask is made of odorless silicon and has a soft seal to enhance patient acceptance and to minimize leakage between the patient's face and the mask. Two ventilation holes 6 mm in diameter are located symmetrically with respect to the central sagittal plane in the lower part of the mask (Figure 1). The dead space volume of the facemask was determined by pressing it onto the surface of a fabricated infant face, Sophia Anatomical Infant Nose Throat (SAINT) model [9] with a defined slight contact force of 0.6 N (about equivalent to the weight of an apple), sealing the ventilation holes and then filling the mask with water. The volume of the water in the mask was considered the dead space of the mask [10]. The dead space volume of the PARI SMARTMASK^®^ Baby was measured as 33 mL.

### 2.2. Simulated Infant Breathing and Nasal Model Conditions

A baby with a weight of 2.5 kg, representing the size of a small newborn infant, was modeled in two ways: a normal breathing pattern and a distressed breathing pattern. The normal breathing pattern was defined as a respiratory rate of 60 breaths per minute, (representing the upper limit of normal for an infant of 0–3 months of age) with a tidal volume (volume of exhaled gas) of 15 mL. The respiratory distress pattern was modeled to mimic the same sized infant with bronchiolitis and was defined as a rate of 120 breaths per minute and a tidal volume of 13.3 mL. For both breathing patterns, the mass fraction of CO_2_ in the modeled exhaled air was fixed at 5%, while outside the mask the atmosphere (see Section 5.4) was considered to have a CO_2_ mass fraction of 0%.

The simulated nasal opening was a cylindrical shape with an internal diameter of 3 mm. The dimensions were derived from the SAINT model and can reasonably be assumed to represent those of human infants. In infants, the choana and the nasal valve of the nasal passage represent the two major bottlenecks through which air must pass. The normal dimensions of the infant nose have been evaluated by CT scan in healthy infants [11].

 The choanal aperture exceeded 3 mm in diameter in all 49 infants (including newborns) without recognized nasal obstructive conditions. Furthermore, a choanal aperture of 3 mm appears to be a reasonable minimal assumption for all infant age groups, including newborns, because choanal aperture is only slightly affected by infant age [11]. During RSV infection, it is expected that mucosal inflammation would decrease the dimensions of the nasal passages, therefore, the 3 mm simulated nasal opening is a reasonable dimension to model an RSV-infected infant.

## 3. One-Dimensional Modeling

### 3.1. Effect of CO_**2**_ Diffusion

When using a one-dimensional approach of investigation, the effects of shear, heat conduction, and diffusion cannot be included. It was therefore necessary to establish whether diffusion effects could be neglected. Thus, an order-of-magnitude analysis based on a one-dimensional and simplified model of the PARI SMARTMASK^®^ Baby was made using the Fick law stating that the diffusive flux is proportional to the concentration gradient [12, 13] and applying binary diffusion coefficients. The concentration gradient of CO_2_ was considered to exist over a 5 mm span between the exhaled gas (with 5% CO_2_) and air outside the mask (no CO_2_). This assumption is likely too high, because the area of the concentration gradient is likely to be larger. Even with this conservative assumption, the mass flow due to diffusion was more than two orders of magnitude smaller than the convected airflow; hence, the diffusion of CO_2_ was less than 1% of the airflow. Therefore, the effects of diffusion were not included in the one-dimensional modeling.

### 3.2. Evaluation of One-Dimensional Model

**Figure 2:**
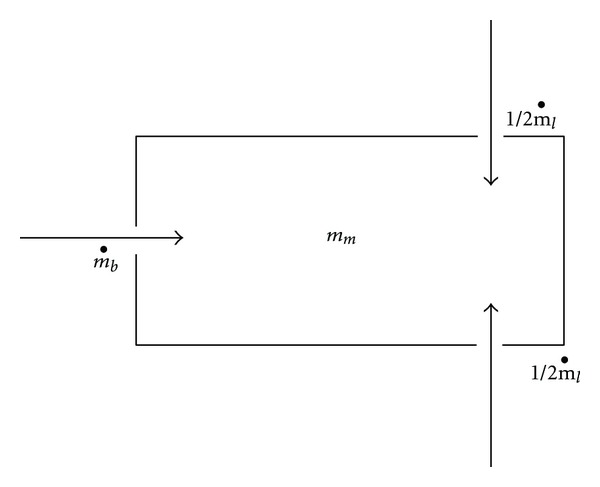
One-dimensional (approximate) model of mask. *m*_*b*_: mass flow of the inhaled and exhaled gas by the baby, *m*_*m*_: mass inside the mask, *m*_*l*_: mass flow of the gas exchanged via the open ventilation holes of the PARI SMARTMASK^®^ Baby. The dot over the *m* denotes the deviation over time (*dm*/*dt*). The direction of the arrows indicates the direction of the mass flow in the one-dimensional, simplified analysis. If the value of the function is positive, the flow goes in the direction of the arrows (into the mask) and if the value of the function becomes negative, the flow goes out of the mask.

Because infants are obligate nosebreathers, the one-dimensional model was set up essentially as a box with one opening representing the nose and two holes of the mask (Figure 2). The Bernoulli equation [14, 15] was applied, with incompressible flow with perfect mixing considered. The simulated patient's in-/exhalation drove the system (pump), and the holes for ventilation controlled the outflow (throttle). The arrows in the figure indicate the positive direction for construction of the mathematical equations. Both directions of mass (air) flow are considered within the model during the full respiratory cycle. The equations were integrated explicitly.

**Figure 3:**
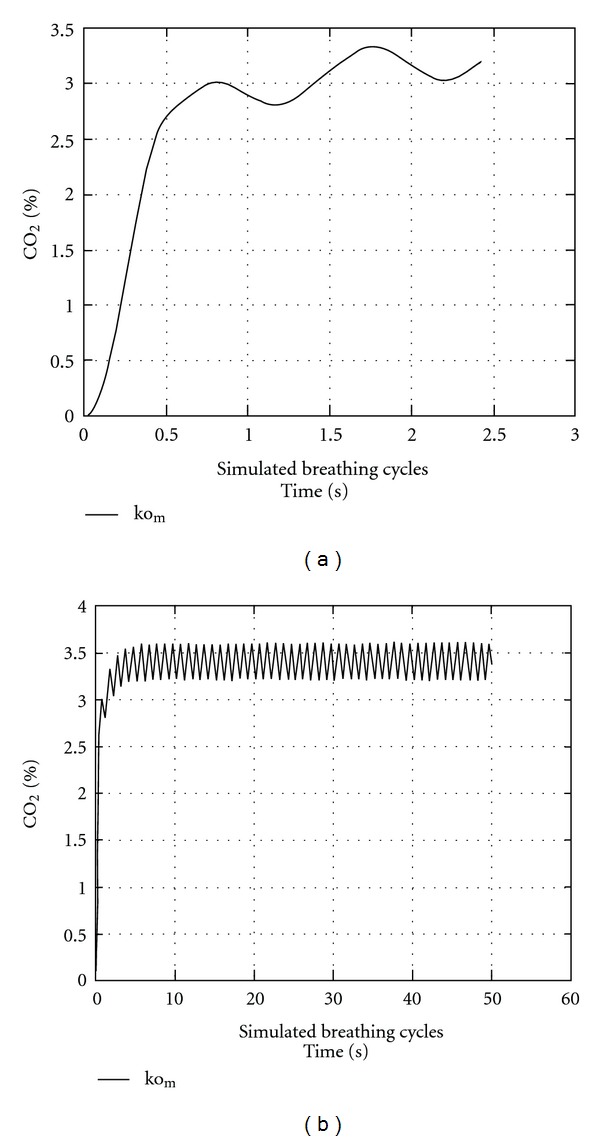
CO_2_ concentrations predicted by one-dimensional model. (a) The % CO_2_ concentrations averaged within the total volume of the mask were modeled over the first three breathing cycles (simulated breaths). (b) The % CO_2_ concentrations were modeled over the first 50 breathing cycles (simulated breaths) to show that steady state is reached early. Both (a) and (b) were modeled using the “normal” infant respiratory pattern (60 breathing cycles/min).

Figure 3(a) shows the evolution of the CO_2_ concentration over five respiratory cycles. A sudden increase in CO_2_ occurred during the first cycle, and then it oscillated between 3.0% and 3.5%. To show that the cycle limit is reached early, 50 cycles were considered and are shown in Figure 3(b), with the conclusion that the steady state is reached after about 10 respiratory cycles. This steady state produces a maximum concentration of CO_2_ in the range of 3.5% averaged within the total volume of the mask. A phase shift in CO_2_ concentration with respect to the beginnings of each respiratory cycle is observed. This is explained by the residual volume of the mask compared to the relatively small tidal volume of the simulated infant. Once the exhaled gas is mixed within the mask, the influence of ambient air during inhalation is delayed.

## 4. Three-Dimensional Modeling

We also performed a computational fluid dynamics (CFDS) analysis to simulate the internal flow within the PARI SMARTMASK^®^ Baby with the objective of evaluating CO_2_ concentration. CO_2_ is metabolically generated, is exhaled, accumulates within the mask, and then is again inhaled into the lungs. An exact CFD solution of the conservation equations (Navier-Stokes equations) provides the information required to evaluate the concentrations of CO_2_ within the mask.

### 4.1. Computational Tools

The computation was performed using the FLUENT software version 6.3.26 commercial CFD solver (FLUENT 6.3 User's Guide, Fluent Inc., now ANSYS Inc., Canonsburg, PA, USA). It solves the conservation equations of mass and momentum (Navier-Stokes equations) iteratively by using a pressure-based, finite volume algorithm. The numerical grid required for the simulation was constructed as an unstructured grid using the GAMBIT software version 2.3.16 grid generator (GAMBIT 2.3 User's Guide, Fluent Inc., now ANSYS Inc., Canonsburg, PA, USA). Flow visualization and postprocessing were carried out with TECPLOT 360 2008 (Tecplot 360 2010 User's Manual, Tecplot Inc., Bellevue, Washington, USA). 

## 5. Results

### 5.1. Computational Model and Domains

**Figure 4:**
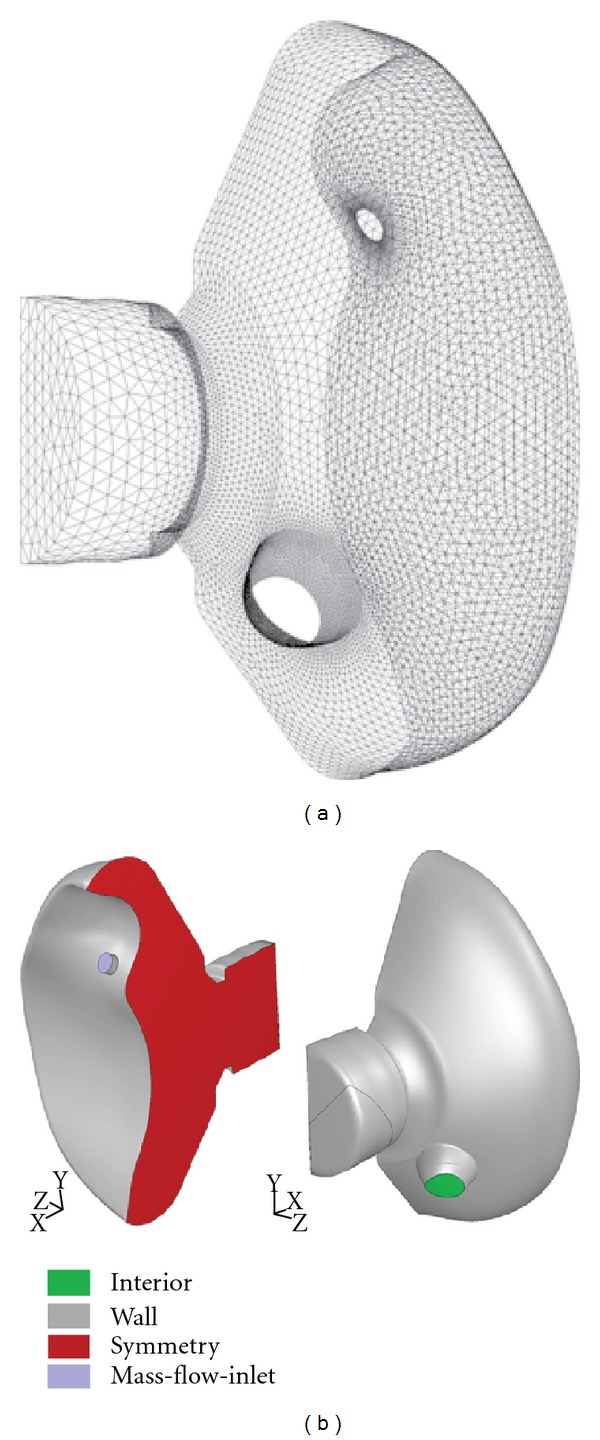
Numerical interior surface grid and boundary conditions of the PARI SMARTMASK^®^ Baby. (a) The grid represents one half of the mask volume, which has been cut and exposed down the sagittal line of symmetry. One half of the infant's face, including the nostril, is also represented. This volume represents the dead space for the three-dimensional modeling. (b) The boundary conditions are represented by different colors.

A solid geometrical 3-dimensional model (computer-assisted design, CAD) of the mask was necessary to generate the numerical grid (Figure 4(a)). Because only the internal flow field was of interest for the computations, the external mask surfaces were removed to simplify the grid generation procedure.

The computational domain is structured as follows. Because the mask is symmetrical with respect to the central sagittal plane, only one half of the mask was considered for the computation (Figure 4). In order to accurately reproduce the air stream flowing into the mask through the inlet opening, a cylindrical external volume (not shown) was attached to the mask in such a way that the volume's axis coincided with the axis of the inlet opening. The volume's dimensions were selected to be sufficiently large to avoid an effect of the external volume boundary on the flow. In addition, the back of the mask domain was modeled to reproduce that part of an infant's face contained within the internal dimensions of the mask (Figure 4).

### 5.2. Numerical Grid

The computational domain was meshed with about 400,000 tetrahedral cells of different sizes. The cell size was varied with the supposed values of flow property gradients as well as with the required accuracy of flow-field solution. A sketch of the numerical grid used for the computation is depicted in Figure 4(a). Note that only the inner surface of the mask itself is shown. Moreover, the main opening (connector where the mask is attached to the nebulizer's mixing chamber) was modeled as closed (Figure 4(b)) so as to provide the worst-case scenario (no air volume coming from the aerosol plume itself).

### 5.3. Numerical Simulation Features

Two infant respiratory flow patterns were computed and analyzed, representing normal breathing and respiratory distress. Both breathing patterns were unsteady (time-dependent) flow simulations considered appropriate for a 0–3-month-old infant.

The flow was assumed to be incompressible for both patterns. This supposition is valid here because the flow Mach number (about 10^−3^) is much less than the theoretical value at which the flow compressibility is significant. Similarly, the flow can be considered to be laminar as the Reynolds number based on the inlet opening diameter is much less than the critical value of 2300 (Re_d_ *≈* 200 for inlet opening diameter). Additionally, the flow was taken to be isothermal at a temperature of 288°K, as heat transfer is unlikely to significantly affect CO_2_ concentration.

The pressure-based segregated approach was used as a numerical method to solve the set of equations governing gas dynamics. In this method, we used a solution algorithm where the equations, namely, the momentum equations, the pressure-correction equation, and the equations of molecular gas species transport, are solved sequentially in an iterative manner. In order to provide a more accurate numerical solution, second-order discretization of the equations was applied in both space and time.

### 5.4. Boundary Conditions

The types of boundary conditions needed to numerically solve the governing equations are depicted in Figure 4(b). The conditions are indicated in terms of the FLUENT solver (ANSYS Inc., Canonsburg, PA, USA). The ambient atmospheric air conditions were set far away from the mask's holes in order to ensure an orifice flow that was free to establish itself physically.

The respiratory cycles were simulated with a sine function that modeled a variation of the air mass flux in the nostril with time. Using the given definitions for normal (60 breaths/min, 15 mL tidal volume) and distressed (120 breaths/min, 13.3 mL tidal volume) breathing, the form of the function was determined for both breathing cases such that air mass conservation was provided. This relation is formulated as an area-specific mass flow of *m* = (1/2)*A*/*S*sin⁡(*ωt*), where *A* = 57.7 × 10^−6^ kg/sec and *ω* = 2*π* rad/sec for normal breathing, *A* = 102.4 × 10^−6^ kg/sec and *ω* = 4*π* rad/sec for quickened breathing, *t* is time in seconds, and *S* = 7.0975 × 10^−6^ m^2^ is the nostril opening's cross-sectional surface area. The factor of 1/2 is to take into account the symmetry of the mask. In this equation, the air mass flux *m* is therefore in kg/m^2^·sec.

Other boundary conditions were assumed to be steady, namely, a static temperature of 288°K and a static pressure of 101325 Pascals (Pa). The mass fraction of CO_2_ in the expired air was taken to be 5%.

### 5.5. CFD-Simulated CO_2_ Concentrations from Three-Dimensional Modeling

**Figure 5:**
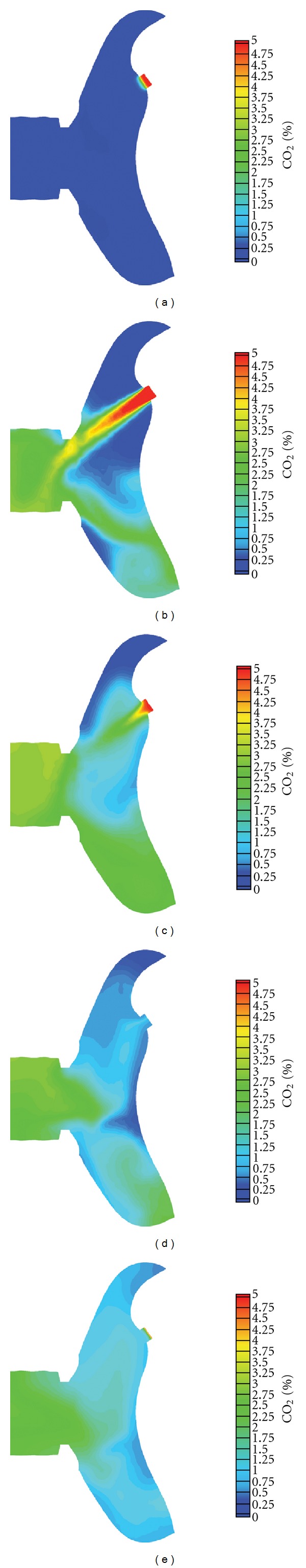
CO_2_ concentration map representing different stages of the first respiratory cycle (normal breathing pattern). A sagittal plane taken parallel to the plane of the center of the infant's nostril is shown at different discrete times within the first modeled respiratory cycle. Blue represents a CO_2_ concentration of zero percent (the modeled concentration of ambient air), and red represents a CO_2_ concentration of 5% (the usual CO_2_ concentration found in exhaled air from a human infant). (a) *t* = 0.005 sec; (b) *t* = 0.25 sec; (c) *t* = 0.5 sec; (d) *t* = 0.75 sec; (e) *t* = 1 sec.

**Figure 6:**
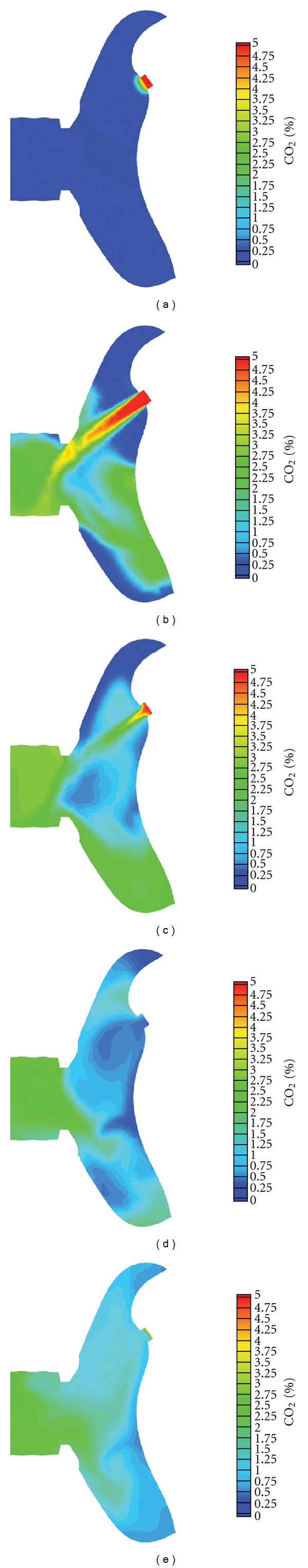
CO_2_ concentration map representing different stages of the first respiratory cycle (distressed breathing pattern). This figure is the same as Figure 5 except that the respiratory cycle takes half as long because the respiratory rate is modeled at 120/min. The individual figures represent the same divisions within the respiratory cycle as in Figure 5. (a) *t* = 0.005 sec; (b) *t* = 0.125 sec; (c) *t* = 0.25 sec; (d) *t* = 0.375 sec; (e) *t* = 0.5 sec.

The CO_2_ distributions were simulated using computational fluid dynamics (see Section 2). Figures 5 and 6 demonstrate simulated CO_2_ concentrations obtained for the first breathing cycle in normal and distressed breathing, respectively. The profiles were taken parallel to the sagittal plane at a distance of *z* = 4.3 mm (which passes through the center of the nostril). They show the distribution of CO_2_ concentration (mass fraction, % CO_2_) as a function of time within a single respiratory cycle.

The first profiles (Figures 5(a) and 6(a)) show CO_2_ mass fractions after the first time interval of the computations (0.005 sec) and are virtually the initial conditions of the CFD simulation. In Figures 5(b) and 6(b), the distribution of CO_2_ mass fractions is shown at a point in time when maximum mass exhaled flows are achieved through the nostril. The exhaled air jet and its interaction with the mask walls are clearly noticeable. As expected, the maximum CO_2_ concentration occurs within the center of the jet. Subsequent profiles are shown for the times of 0.5 and 0.25 sec for normal and distressed breathing, respectively (Figures 5(c) and 6(c)). This corresponds to one-half of the breathing cycle for normal and distressed breathing patterns, respectively, where the zero air mass flow occurs through the nostril.

Figures 5(d) and 6(d) show the profiles of CO_2_ concentration when the maximum negative mass flows are achieved, that is, at the peak of inhalation. Despite the fact that the maximum quantity of ambient air is received at this moment through the inlet opening, it can be noticed that the inhaled air is nevertheless impure, with a CO_2_ concentration of up to 1.05%.

Finally, the last profiles (Figures 5(e) and 6(e)) show the distribution of CO_2_ at the end of the breathing cycle, when zero mass flow takes place.

**Figure 7:**
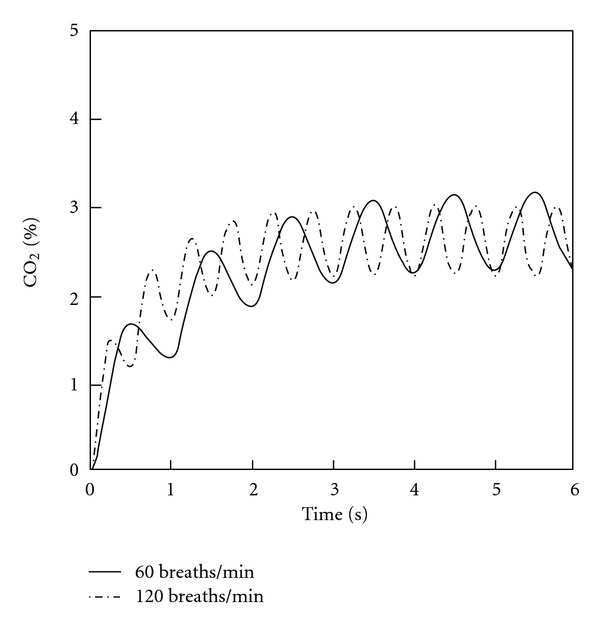
CO_2_ concentrations averaged over the entire volume of the mask during successive respiratory cycles. Both normal and distressed breathing patterns are shown.

**Figure 8:**
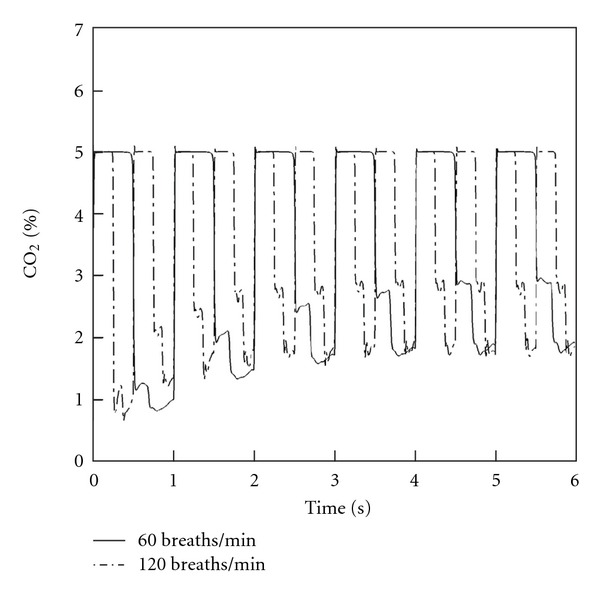
CO_2_ concentrations averaged at the level of the nostril during successive respiratory cycles. Both normal and distressed breathing patterns are shown.

Averaged mass-weighted % CO_2_ concentrations occurring in the internal volume of the mask and at the nostril entrance are shown in Figures 7 and 8, respectively. The initial twelve cycles of breathing are demonstrated for both normal and distressed infant breathing patterns.

A steady increase in concentration is obtained during the initial 4-5 cycles, after which a steady periodical variation of CO_2_ concentration occurs. This steady state occurs both within the mask's interior and at the nostril entrance. The maximum average CO_2_ concentrations achieved in the mask were 3.2% and 3% for the normal and distressed breathing patterns, respectively (Figure 7). When integrated over the inspiratory phase of the simulated breathing cycle, the average CO_2_ concentrations inhaled at the level of the nostril after steady state had been achieved were 2.24% and 2.26% for the normal and distressed breathing patterns, respectively (Figure 8).

## 6. Discussion

Masks to efficiently and safely deliver aerosolized medications to infants are needed. Efficient delivery of aerosols requires that the mask be relatively tight fitting so as to minimize leakage of the aerosol away from the infant. However, a tight-fitting mask may increase the possibility of CO_2_ retention and rebreathing of exhaled air by the patient unless specific design considerations are sought to combat the problem. CO_2_ retention is not as problematic with jet nebulizers because they generate aerosols by a continuous stream of air, which also washes out exhaled CO_2_ within a mask. New nebulizer technologies are designed to increase delivery efficiency of drug to the lung but do not employ an air stream to generate the aerosol, and a tighter fitting face mask might further improve this delivery efficiency. However, the lack of a driving air stream combined with a tight-fitting mask may be problematic because of rebreathing of exhaled CO_2_.

We designed a new infant facemask for efficient aerosol delivery of an RSV antiviral agent and evaluated its CO_2_ retention properties. To our knowledge, this is the first report of such evaluation of an infant mask. Our analysis consisted of modeling CO_2_ retention and rebreathing under conditions paralleling those encountered clinically during both a typical mild and a typical severe RSV bronchiolitis illness. Furthermore, our design modeled an age range of patients at high risk for RSV lower respiratory tract infection by actually using an age-appropriate infant's face shape [9].

Our simulation model showed that CO_2_ concentrations within the mask reached steady state within approximately 4-5 breaths and did not accumulate thereafter. Mean CO_2_ concentrations inspired into the nostrils of the simulated infant during the inspiratory part of the respiratory cycle and after steady state concentrations were reached were 2.24% and 2.26% for the normal and distressed infant breathing patterns, respectively.

The medical and physiological effects on an infant of breathing this concentration of CO_2_ deserve discussion. Although the body requires oxygen for metabolism, low oxygen levels do not stimulate breathing significantly. Rather, breathing (ventilation) is stimulated by increased CO_2_ levels in the blood. CO_2_ is the classic respiratory stimulus. Increasing the CO_2_ in inspired air quickly increases the concentration of CO_2_ in the blood, which, in turn, rapidly stimulates chemoreceptors in the central nervous system. These central nervous system chemoreceptors then send neural signals to the muscles of respiration, causing breathing volumes and frequency to increase. Breathing faster and deeper blows off more CO_2_, which leads to a reduction of the blood CO_2_ and consequently a reduction in the concentration of exhaled CO_2_. Through these neural feedback loops, which are present at all ages, the ventilation is quickly adjusted so as to maintain the body's pH and blood CO_2_ concentrations within very narrow ranges. These respirations are altered rapidly, within seconds after central pH changes occur. Whether or not these homeostatic responses are sufficient, given the alterations produced by the mask, is what predicts this major component of mask safety.

Lung volumes per body surface area are the same in newborns and adults [16]. This also applies to the physiological dead space volume per body weight. The relative dead space of newborns and adults is equally 2.2 mL/kg [16, 17]. Because of their small size, the absolute dead space of newborns is much smaller than that of adults. For a 2.5 kg newborn the dead space is approximately 5.5 mL, whereas for a 70 kg adult the dead space is around 150 mL. Therefore, an increase of dead space by a certain fixed amount has a potentially greater impact on newborns than on adults because it raises the ratio of dead space volume to tidal volume (*V*_*D*_/*V*_*T*_) disproportionally. A higher (*V*_*D*_/*V*_*T*_) ratio causes a decrease of alveolar ventilation. As a consequence, the reinhalation of exhaled CO_2_-rich air within the mask could lead to a higher partial CO_2_ pressure (pCO_2_) in the blood [17]. To avoid this, the dead space of infant masks and the reinhalation of exhaled CO_2_ should be held to acceptable levels.

The respiratory response to increasing dead space of pediatric anesthesia equipment has been studied [18]. Additionally, the respiratory response to increasing concentrations of inspired CO_2_ has been studied in infants, children, and adults, and, in the awake state, do not differ with age [19, 20]. Infants have been shown to increase their minute ventilation (total volume of expired air within one minute) by 16% when inspiring 2.22% CO_2_, and by 34% when inspiring 3.71% CO_2_ [20]. Neonates accomplish this by simply increasing their tidal volume, with no need to increase their respiratory rate or alter their breathing pattern [20]. Moreover, this increase in minute ventilation begins rapidly (within 30 sec) in infants rebreathing elevated concentrations of CO_2_, and the end-tidal CO_2_ concentrations measured at the nose also reached steady state within this same rapid time frame [20]. Even newborns a few days old were studied, and when forced to breath 2, 4, and 6% CO_2_, they had the same magnitude of increase in ventilation as do adults [19].

Thus, by extrapolation, since the PARI SMARTMASK^®^ Baby exposes the nose to inspired mean CO_2_ concentrations of 2.2-2.3%, the mask could be expected to result in only a slight increase in minute ventilation of approximately 15–20% [20]. Because infants normally maintain CO_2_ homeostasis by changing their minute ventilation by as high as 400–800%, a 15–20% increase would be expected to have no clinical impact except in infants with severe lung disease who were nearing requirement for mechanical ventilation. Furthermore, adapting a supplemental oxygen input system onto the existing eFlow^®^ aerosol delivery device would serve both to increase oxygen delivery to the neonate and to wash out some exhaled CO_2_ from the mask, thus further decreasing the potential rebreathing of CO_2_.

Inspiring increased concentrations of CO_2_ (up to 4%) does not alter other important aspects of infant pulmonary physiology, such as, dynamic compliance and total pulmonary resistance [20]. Since infants with RSV infection (bronchiolitis and pneumonia) have increased airway resistance due to edema and secretions narrowing their upper and lower airways, it is imperative not to depress inspiratory drive, the negative pressure that infants generate during inspiration. Importantly, inspiratory drive was actually increased by 75% in those infants breathing higher concentrations of CO_2_ [20]. This would serve to maintain or even aid the movement of air through upper and lower airways potentially narrowed by RSV-induced secretions and or edema.

There are limitations as to the model's ability to account for physiologic variables occurring in infants themselves. The model used a sine wave function for the respiratory cycle. The minute ventilation was modeled appropriately. However, actual peak inspiratory and expiratory flow rates in adults and infants are greater than those modeled by the sine wave. These higher inspiratory and expiratory peak flow rates would likely serve to increase the turbulence within the mask, thereby increasing the mixing of air and increasing the exchange of air through the ventilation holes in the mask. This would tend to decrease the inhaled CO_2_ concentrations below those calculated here. Another limitation is that the model did not account for the normal physiologic response to rebreathing CO_2_. Homeostatic mechanisms previously discussed might tend to increase both respiratory rate and peak inspiratory/expiratory flow rates. These effects would further increase mask turbulence and air exchange through the ventilation holes. Thus our model tends to overestimate the concentrations of CO_2_ using the mask. Indeed these kinds of overestimations have been experienced before. The volume of dead space within anesthesia masks has been shown to have minor clinical significance [21]. Additional supporting evidence comes from *in vivo* experiments showing that a mask's internal volume measurement markedly overestimates its physiologically produced functional dead space [22].

In conclusion, a mask has been designed (the PARI SMARTMASK^®^ Baby) to efficiently deliver aerosols to infants. We evaluated the CO_2_ retention properties of this mask using models simulating normal infants and infants with mild and severe RSV infection. The mask appears to cause minimal CO_2_ retention and rebreathing. The concentrations of CO_2_ modeled to occur within the mask (and more importantly, inspired into the nostrils) are within ranges previously evaluated in infants and children. This study did not address infants whose existing lung disease is so severe as to already be causing abnormal elevations in blood CO_2_ concentrations. Infants with normal and diseased lungs should tolerate the mask when used to intermittently deliver therapeutic aerosols over brief time frames.
